# Identification and prediction model of placenta-brain axis genes associated with neurodevelopmental delay in moderate and late preterm children

**DOI:** 10.1186/s12916-023-03023-1

**Published:** 2023-08-26

**Authors:** Yumin Zhu, Yimin Zhang, Yunfan Jin, Heyue Jin, Kun Huang, Juan Tong, Hong Gan, Chen Rui, Jia Lv, Xianyan Wang, Qu’nan Wang, Fangbiao Tao

**Affiliations:** 1 Medical School, Nanjing University, Nanjing, Jiangsu China; 2 Department of Maternal & Child and Adolescent Health, School of Public Health, MOE Key Laboratory of Population Health Across Life Cycle, Anhui Provincial Key Laboratory of Population Health and Aristogenics, Anhui Medical University, Hefei, Anhui China; 3 MOE Key Laboratory of Bioinformatics, Center for Synthetic and Systems Biology, School of Life Sciences, Tsinghua University, Beijing, China; 4 Department of Toxicology, School of Public Health, Anhui Medical University, Hefei, Anhui China

**Keywords:** Moderate and late preterm, Neurodevelopment, Birth cohort, Placenta, RNA biomarker, Transcriptome, Machine learning

## Abstract

**Background:**

Moderate and late preterm (MLPT) birth accounts for the vast majority of preterm births, which is a global public health problem. The association between MLPT and neurobehavioral developmental delays in children and the underlying biological mechanisms need to be further revealed. The “placenta-brain axis” (PBA) provides a new perspective for gene regulation and risk prediction of neurodevelopmental delays in MLPT children.

**Methods:**

The authors performed multivariate logistic regression models between MLPT and children’s neurodevelopmental outcomes, using data from 129 MLPT infants and 3136 full-term controls from the Ma’anshan Birth Cohort (MABC). Furthermore, the authors identified the abnormally regulated PBA-related genes in MLPT placenta by bioinformatics analysis of RNA-seq data and RT-qPCR verification on independent samples. Finally, the authors established the prediction model of neurodevelopmental delay in children with MLPT using multiple machine learning models.

**Results:**

The authors found an increased risk of neurodevelopmental delay in children with MLPT at 6 months, 18 months, and 48 months, especially in boys. Further verification showed that *APOE* and *CST3* genes were significantly correlated with the developmental levels of gross-motor domain, fine-motor domain, and personal social domain in 6-month-old male MLPT children.

**Conclusions:**

These findings suggested that there was a sex-specific association between MLPT and neurodevelopmental delays. Moreover, *APOE* and *CST3* were identified as placental biomarkers. The results provided guidance for the etiology investigation, risk prediction, and early intervention of neurodevelopmental delays in children with MLPT.

**Supplementary Information:**

The online version contains supplementary material available at 10.1186/s12916-023-03023-1.

## Background

The World Health Organization defines preterm birth as delivery before 37 weeks of gestation and is further classified according to the gestational age of delivery: extremely preterm birth (< 28 weeks), very preterm birth (28–31^+6^ weeks), and moderate and late preterm birth (MLPT) (32–36^+6^ weeks) [1]. Extremely preterm birth and very preterm birth have attracted much attention due to high mortality and morbidity [2–5]. However, in recent years, studies have found that children with MLPT are also at greater risk of neurobehavioral developmental delays compared with full term (FT) children [6]. MLPTs account for approximately 84% of preterm births globally, and the dramatic increase in preterm birth rates in recent decades is largely attributable to the growth in the number of MLPTs [7, 8]. Therefore, MLPT is a global public health problem, and its impact on human health, population quality, and national economy cannot be ignored.

Abnormal intrauterine environment is not suitable for fetal growth and development and may lead to the occurrence of MLPT. The placenta is not only a maternal–fetal barrier but also a maternal–fetal link bridge between endogenous development and postnatal growth and development [9–12], which plays a key role in maintaining a suitable intrauterine environment. Therefore, the placenta is an important organ for understanding the intrauterine regulatory mechanism of neurodevelopmental outcomes, and the concept of the “placenta-brain” axis was born [13], which has become a potential source of early identification of high-risk infant biomarkers. Recent studies have found that lots of abnormally regulated genes in the placenta affect fetal brain development; these genes are defined “placenta-brain axis (PBA) genes,” including genes in pathways related to neurotransmitter synthesis and secretion, immunity and inflammation, and cell surface receptor signaling [14, 15].

In this study, we first investigated the association between MLPT and neurodevelopmental delays in children based on the prospective birth cohort MABC. Secondly, we screened out the abnormally regulated PBA-related gene sets in MLPT children’s placentas and verified them in an independent cohort, providing a basis for placental regulation in MLPT children with an increased risk of neurodevelopmental delays. Finally, we further identified sex-specific RNAs associated with neurodevelopmental outcomes in the placentas of MLPT children through RT-qPCR and established a risk prediction model based on these RNA biomarkers using machine learning methods. This study provides guidance for the etiology investigation, risk prediction, and early intervention of neurodevelopmental delays in children with MLPT.

## Methods

### Study design and population

This study was based on Ma’anshan Birth Cohort (MABC), which is a longitudinal study of health during early life period in Ma’anshan, Anhui, China. The MABC was recruited from May 2013 to September 2014, with women from the Ma’anshan Maternal and Child Health Hospital. The inclusion criteria for pregnant women were as follows: (1) ≥ 18 years old and ≤ 14 gestational weeks; (2) willing to have antenatal checkups and childbirth in Ma’anshan Maternal and Child Health Hospital; (3) had no serious physical diseases or serious mental illness and could complete the questionnaires independently; (4) willing to participate in follow-ups during childhood. A total of 3474 pregnant women from MABC study were eligible. The study protocol has been approved by the Ethics Committee of Anhui Medical University (No. 20131195). All participants provided written informed consent. A detailed description of the cohort information and inclusion and exclusion criteria has been published elsewhere [16].

The China National Birth Cohort (CNBC) was used as an independent sample source to verify the differential expression of PBA-related genes. The CNBC study conducted in Ma’anshan, Anhui, China, is a prospective birth cohort study recruited from May 2017 to September 2018. The inclusion criteria for pregnant women were as follows: (1) Chinese residents of Ma’anshan city; (2) ≥ 18 years old and ≤ 14 gestational weeks; (3) conceived naturally; (4) willing to have antenatal checkups and childbirth in Ma’anshan Maternal and Child Health Hospital; (5) willing to participate in follow-ups during childhood. A total of 1508 women were recruited from the Ma’anshan Maternal and Child Health Hospital. Details of the inclusion exclusion criteria have been published previously [17].

### Children’s neurodevelopment assessment and definition

Children’s neurodevelopmental behavior was assessed by the Ages and Stages Questionnaire of China (ASQ-C) at the age of 6 months, 18 months, and 48 months based on the responses filled in by the children’s parents or guardians. The ASQ-C was translated from the ASQ with necessary cross-cultural adaptations for China [18], which contains a series of 30 questions representing five domains or developmental areas: gross motor skill domain, fine motor skill domain, communication domain, personal-social skill domain, and problem solving domain. The sum of the scores from the 6 questions in each domain provides the final score [19]. The maximum score of each domain was 60, a score that was 1 SD (standard deviation) below the mean of each domain was used as the principal cutoff point in the present study, and children with scores in each domain below 1 SD relative to the mean were defined as having SDD (suspected developmental delay) [20].

### Confounders and covariates

According to previously published studies [21] and our univariate analysis results, we obtained following confounders and covariates: maternal information about the age at children’s birth, pre-pregnancy body mass index (BMI), feeding patterns at 6 months, monthly income of family per capita, educational level, smoking status during pregnancy, pregnancy-related anxiety, and children’s information about sex.

Information on monthly income of family per capita, educational level and smoking during pregnancy, feeding patterns at 6 months, and pregnancy-related anxiety were obtained from children’s mothers in the structured self-administered questionnaires. Information on maternal age at child’s birth and child sex were extracted from medical records. Maternal pre-pregnancy BMI [BMI = weight (kg)/height (m)^2^] was measured by doctors at the time of the first prenatal checkup.

### Placenta samples collection

Placental tissue samples were collected by trained personnel immediately after delivery. The edge part of the placenta and the area near the umbilical cord were avoided during sample collection. The samples were taken at least 3 cm from the cord insertion and 3 cm from the placental edge. After washing with normal saline, extracted placental lobules without calcified or fascia were dissected and longitudinally cut into four equal-sized sections, each containing both maternal and fetal surfaces. They were then immersed in RNAlater and refrigerated for 4 °C overnight. The next morning, after pouring off the RNAlater fluid, the placental tissue was stored at − 80 °C until use.

### RNA extraction and sequencing

We selected 30 MLPT placentas from the placenta samples collected from the MABC cohort as the case group for RNA sequencing, including 15 male MLPT placentas and 15 female MLPT placentas. In addition, strictly matching the age of pregnant women, pre-pregnancy BMI, and fetal sex, we selected 30 FT placentas as the control group for RNA sequencing. Total RNA was extracted from the placenta using TRIzol® Reagent according the manufacturer’s instructions (Magen). RNA samples were detected based on the A260/A280 absorbance ratio with a Nanodrop ND-2000 system (Thermo Scientific, USA), and the RIN of RNA was determined by an Agilent Bioanalyzer 4150 system (Agilent Technologies, CA, USA). Qualified samples were used for library construction. Paired-end libraries were prepared using a ABclonal mRNA-seq Lib Prep Kit (ABclonal, China) following the manufacturer’s instructions. The mRNA was purified from 1 μg total RNA using oligo (dT) magnetic beads followed by fragmentation carried out using divalent cations at elevated temperatures in ABclonal First Strand Synthesis Reaction Buffer. Subsequently, first-strand cDNAs were synthesized with random hexamer primers and reverse transcriptase (RNase H) using mRNA fragments as templates, followed by second-strand cDNA synthesis using DNA polymerase I, RNAseH, buffer, and dNTPs. The synthesized double stranded cDNA fragments were then adapter-ligated for preparation of the paired-end library. Adaptor ligated cDNA were used for PCR amplification. PCR products were purified (AMPure XP system) and library quality was assessed on an Agilent Bioanalyzer 4150 system. Finally, sequencing was performed with an Illumina Novaseq 6000 /MGISEQ-T7 instrument.

### Bioinformatics analysis of RNA-seq data

The data generated from Illumina platform were used for bioinformatics analysis. Raw data of fastq format were firstly processed through fastp (https://github.com/OpenGene/fastp). In this step, remove the adapter sequence and filter out low quality (low quality, the number of lines with a string quality value less than or equal to 25 accounts for more than 60% of the entire reading), and *N* (*N* means that the base information cannot be determined) ratio is greater than 5% reads to obtain clean reads that can be used for subsequent analysis. Samples that failed quality control (QC) were removed from downstream analysis. Then, clean reads were separately aligned to reference genome with orientation mode using the HISAT2 software (http://daehwankimlab.github.io/hisat2/) to obtain mapped reads. FeatureCounts (http://subread.sourceforge.net/) was used to count the reads numbers mapped to each gene. And then FPKM of each gene was calculated based on the length of the gene and reads count mapped to this gene. We used edgeR package to identify differentially expressed genes (DEGs) between the placentas of MLPT children and FT children [22]. Based on some published literature [23–25], genes were considered statistically significant at |log_2_fold-change|> =|log_2_(1.5)| and FDR < 0.2. To evaluate the biological functions, the computational web servers g:Profiler (https://biit.cs.ut.ee/gprofiler/gost) was used to perform Kyoto Encyclopedia of Genes and Genomes (KEGG) and the gene ontology (GO) pathway analysis for DEGs of interest [26]. GO and KEGG pathway terms with the FDR of the Fisher’s exact test < 0.05 were considered statistically significant.

### Curation of PBA-related genes

A comprehensive search was performed to identify studies from inception to 18 January 2022 that involved PBA genes. The applied search terms were broad, including synonyms and truncations of “placenta,” “neurodevelopment,” “predict,” “biomarker,” “brain,” “multi-omics,” and “disorders.” The selected literature must contain both the terms “placenta” and “brain” and the remaining terms help to filter. We carefully read the collected literature and excluded some of the irrelevant literature, to ensure that the literature cited can provide the PBA genes and PBA pathways. The PBA genes were collected from literature that suggested that abnormalities of these genes in the placenta could cause neurodevelopmental abnormalities in children. The PBA pathway genes were collected from PBA pathways, including neurotransmitter-related pathways such as serotonin, dopamine, and norepinephrine/adrenaline. These neurotransmitters were secreted by the placenta during pregnancy and had an important impact on fetal brain development [13, 27–30]. There were 40 PBA genes and 636 PBA pathway genes, of which 7 genes belong to both PBA genes and PBA pathway genes. Finally, we collected 669 PBA-related genes in total.

### RT-qPCR validation

RNA extraction and RT-qPCR were performed according to the manufacturer’s instructions. Briefly, total RNA was extracted from placenta samples using Trizol (Invitrogen 15,596–026). The concentration and purity of RNA were measured by Denovix spectrophotometer (Thermo Scientifc, Wilmington, DE, USA). Then, RNA was reverse-transcribed using the Evo M-MLV RT-PCR Kit (AG11707). Real-time RT-PCR was implemented after reverse transcription. Expression levels were quantified using a LightCycler 96 Instrument’s realtime PCR system (Roche 05815916001) with a SYBR Green master mix (Yeasen 11201ES08). Primers are shown in Additional file 1: Table S1. Data were exported to Excel; relative gene expression was calculated by the comparative CT method (ΔΔCT) and normalized based on the level of human 18S mRNA. The differences between two groups were analyzed using a two-tailed Mann–Whitney test. Each value reflects the mean ± standard error of the mean (SEM) of at least three different biological replicates.

### Machine learning method for building prediction models

Three machine learning methods including logistic regression (LR), random forest (RF), and decision tree (DT) were used to evaluate the classification performance of the selected features. We used the leave-one-out cross-validation (LOOCV) for model selection. The prediction performance of each model was evaluated by the area under the receiver operating characteristic curve (AUROC). All analyses were performed with python version 3.10.

### Statistical analysis

Descriptive statistics for maternal demographic and obstetric characteristics were described by MLPT using the mean ± standard deviation (SD) or percentage (*n* %). The differences between two groups were analyzed using Student’s *t*-test. Multiple comparisons were performed using ANOVA.

The association between MLPT and neurodevelopmental delay at 6, 18, and 48 months was analyzed separately by children’s sex by using the odds ratio (OR) and 95% confidence interval (CI) in multivariable logistic regression model. The repeated measurement analysis of the association between MLPT and different ASQ domains was calculated by using generalized estimating equations (GEE) analysis. The Mann–Whitney test was used to compare the two groups such as placental mRNA expression and neurodevelopmental delay.

Statistical analyses, including Student’s *t*-test, ANOVA, logistic regression, Mann–Whitney test, and receiver operator characteristic (ROC), were performed by the R software (ver. 4.1.2; R Foundation for Statistical Computing, Vienna, Austria) and SPSS (version 22.0, Chicago, IL, USA) software. The significance threshold was set at a *p*-value < 0.05.

## Results

### Demographics

**Fig. 1:**
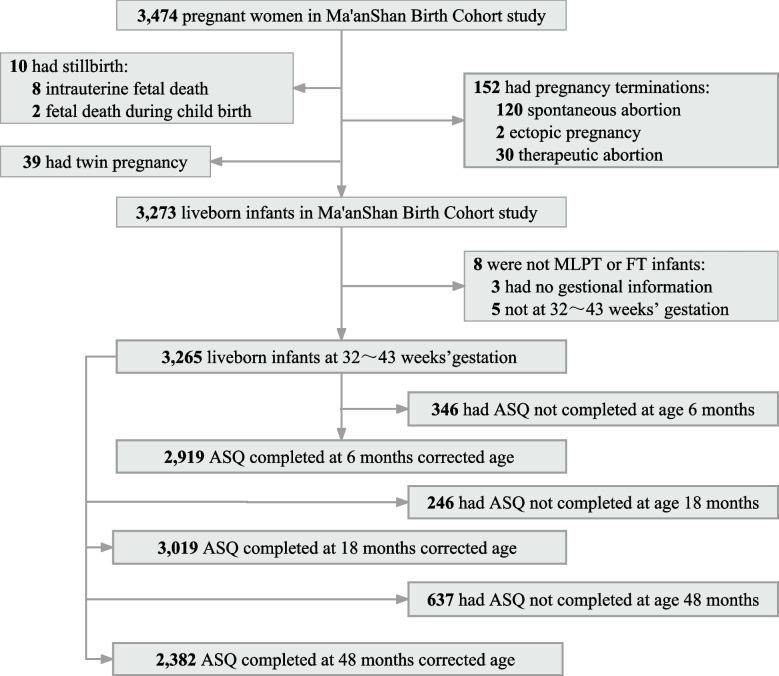
Flowchart of participants through the children’s ASQ-C follow-up

This study was based on Ma’anshan Birth Cohort (MABC). One hundred twenty-nine MLPT infants and 3136 full term (FT) controls were recruited. The average gestational age at delivery was 35.2 weeks (standard deviation [SD], 1.11) for the MLPT group and 39.2 weeks (SD, 1.08) for the FT group. Compared with the FT group, the infants in the MLPT group were more likely to have lower birth weights; the mothers in the MLPT group have greater age at delivery, higher prenatal BMI, higher smoking rates, and higher rates of pregnancy-related complications, such as diabetes mellitus in pregnancy and hypertensive disorder complication pregnancy (Table 1 and Fig. 1).

**Table 1:** Demographic characteristics of the study subjects

**Demographics and reproductive characteristics**	**MLPT group** **(** ***n*** ** = 129)** **Mean (SD)**	**Term group** **(** ***n*** ** = 3136)** **Mean (SD)**	***p*** **-value**
**Maternal age (years)**	27.40 (4.21)	26.60 (3.61)	**0.028**
**Pre-pregnancy BMI (kg/m**^**2**^**)**	21.40 (3.54)	20.60 (2.74)	**0.008**
**Birthweight (grams)**	2640 (465)	3400 (413)	** < 0.001**
**Gestational age at delivery (weeks)**	35.20 (1.11)	39.2 (1.08)	** < 0.001**
	***N*** ** (%)**	***N*** ** (%)**	
**Child sex (male)**	79 (61.2)	1589 (50.7)	**0.024**
**Parity**			0.719
Primipara	116 (89.9)	2775 (88.5)	
Multipara	13 (10.1)	361 (11.5)	
**Education**			0.712
Junior high school or below	29 (22.5)	628 (20.0)	
High school	24 (18.6)	710 (22.6)	
Junior college	42 (32.6)	971 (31.0)	
University or above	34 (26.4)	827 (26.4)

### Associations between MLPT and neurodevelopmental delay

**Fig. 2:**
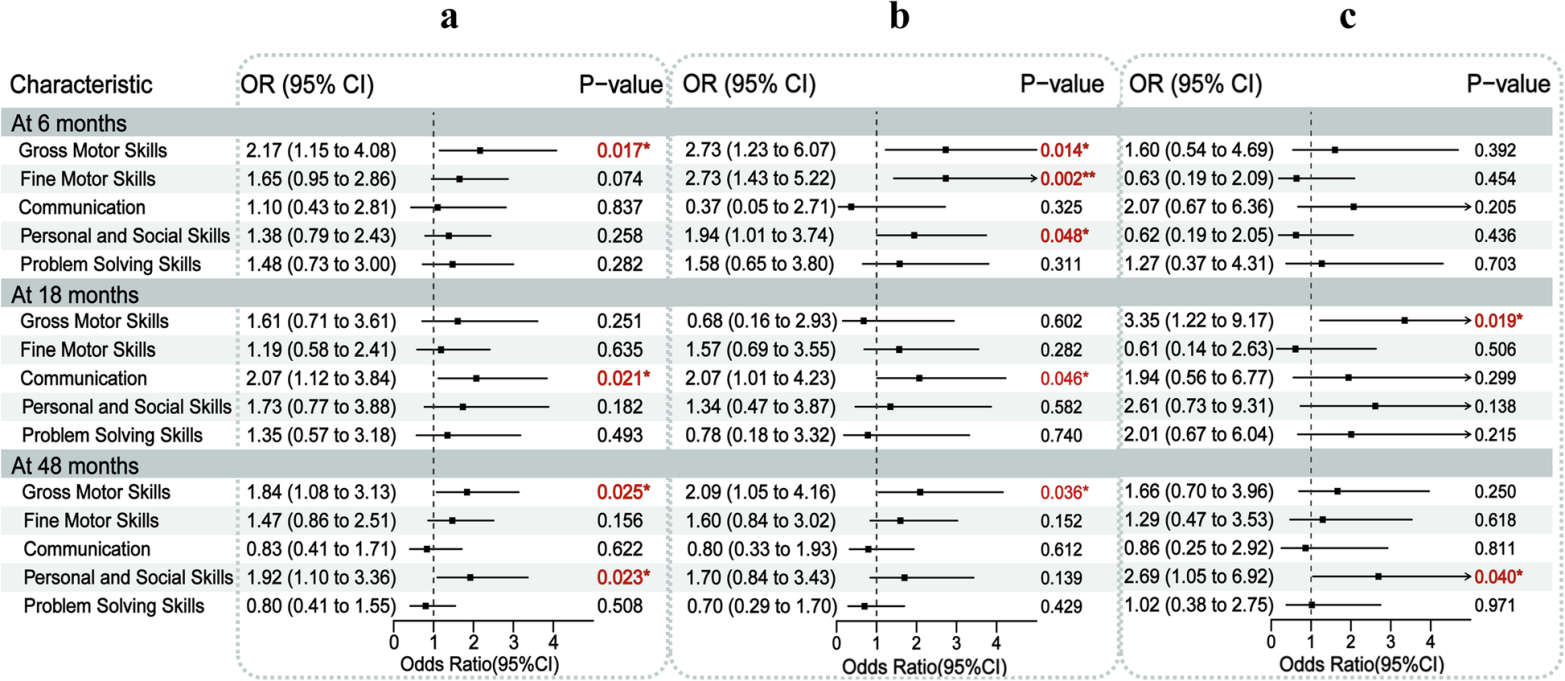
Associations between MLPT exposure and neurodevelopmental delays. **a** All. **b** Male. **c** Female. The model adjusted for characteristics of mothers (maternal age, maternal pre-pregnancy BMI, maternal educational level, monthly income of family per capita, passive smoking status, breastfeeding duration, pregnancy-related anxiety) and children (sex). *p < 0.05, **p < 0.01, multivariate logistic regression analysis. MLPT, moderate and late preterm; BMI, body mass index; ASQ, Ages and Stages Questionnaire; CI, confidence interval; OR, odds ratio

We applied ASQ-C to assess children’s neurodevelopmental behavior at the age of 6, 18, and 48 months, which filled in by the children’s parents or guardians. Then, we evaluated the associations between MLPT and neurodevelopmental outcomes based on ASQ-C data. The age of pregnant women, body mass index (BMI) before pregnancy, educational level of pregnant women, duration of breastfeeding, child sex, per capita monthly income of the family, pregnancy-related anxiety, and smoking status of pregnant women were taken as confounding factors; the development of MLPT children in the gross motor domain at 6 months was significantly delayed (*OR* = 2.17, 95% *CI* = 1.15–4.08, *P*-value = 0.017); the development of MLPT children in communication domain was significantly delayed at 18 months (*OR* = 2.07, 95% *CI* = 1.12–3.84, *P*-value = 0.021); the development of MLPT children in gross motor domain (*OR* = 1.84, 95%*CI* = 1.08–3.13, *P*-value = 0.025) and personal-social domain (*OR* = 1.92, 95% *CI* = 1.10–3.36, *P*-value = 0.023) was significantly delayed at 48 months (Fig. 2a). Stratified by sex, boys with MLPT showed significantly delayed development in the gross motor domain (*OR* = 2.73, 95% *CI* = 1.23–6.07, *P*-value = 0.014), fine motor domain (*OR* = 2.73, 95% *CI* = 1.43–5.22, *P*-value = 0.002), and personal-social domain (*OR* = 1.94, 95% *CI* = 1.01–3.74, *P*-value = 0.048) at 6 months, the communication domain at 18 months (*OR* = 2.07, 95% *CI* = 1.01–4.23, *P*-value = 0.046), and the gross motor domain at 48 months (*OR* = 2.09, 95% *CI* = 1.05–4.16, *P*-value = 0.036) (Fig. 2b). As for girls with MLPT, the development of gross motor domain was significantly delayed at 18 months (*OR* = 3.35, 95% *CI* = 1.22–9.17, *P*-value = 0.019), and the development of personal-social domain was significantly delayed at 48 months (*OR* = 2.69, 95% *CI* = 1.05–6.92, *P*-value = 0.040) (Fig. 2c). Moreover, repeated measurement analysis and multiple testing correction does enhance the reliability of the results, so we calculated the association between MLPT and different domains of ASQ at 6, 18, and 48 months by using generalized estimating equations (GEE) analysis (Additional file 1: Table S2) and reported false discovery rates of different domains and different ages of ASQ respectively calculated by using Bonferroni-Holm method (Additional file 1: Table S3). After adjustment for confounders same as above, we found that the development of MLPT children in the gross motor domain (*OR* = 0.58, 95% *CI* = 0.39–0.85,* P*-value = 0.006) and personal-social domain was significantly delayed (*OR* = 0.61, 95% *CI* = 0.42–0.90,* P*-value = 0.013). Stratified by sex, boys with MLPT showed significantly delayed development in the gross motor domain (*OR* = 0.56, 95% *CI* = 0.33–0.95,* P*-value = 0.033), fine motor domain (*OR* = 0.56, 95% *CI* = 0.35–0.90,* P*-value = 0.018), and personal-social domain (*OR* = 0.60, 95% *CI* = 0.39–0.93,* P*-value = 0.021). No significant result was found between MLPT and neurodevelopmental delay in girls. These results provide more convincing evidence of an increased risk of neurodevelopmental delay in MLPT children, especially in boys.

### Transcriptome-wide analysis of placentas from MLPT and full-term birth

**Fig. 3:**
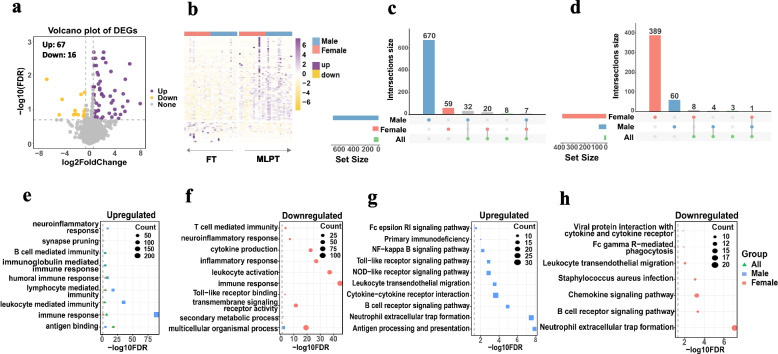
Analysis of differentially expressed genes (DEGs) in placenta samples from MABC study. **a** Volcano plot for all genes in the MLPT infants’ placentas vs FT infants’ placentas. **b** Heatmap of DEGs in placenta samples of MLPT infants and FT infants. **c**, **d** Upset plot of upregulated genes and downregulated genes of both all, male, and female. **c** Upregulated; **d** downregulated. **e**, **f** GO functional annotation clustering analysis of DEGs. **g**, **h** KEGG pathway analysis

To examine whether alterations in the placental transcriptome are associated with an increased risk of neurodevelopmental delay in children with MLPT, we profiled RNA-seq to identify dysregulated genes in the placentas of MLPT infants (Additional file 1: Table S4). Considering sex specificity, we divided the data into three groups for analysis, namely “all,” “male,” and “female” groups. In total, 67 upregulated genes and 16 downregulated genes were identified between MLPT and FT in the all group (Fig. 3a, b). Seven hundred nine upregulated genes and 65 downregulated genes were identified between MLPT and FT in the male group; 86 upregulated genes and 398 downregulated genes were identified between MLPT and FT in the female group (Additional file 1: Table S5). There were only a few common DEGs among the all, male, and female groups (Fig. 3c, d), which reflects that the relevance of placental gene expression to MLPT is sex-specific and provides potential clues for the sex specificity in incidence and risks of neurodevelopmental delays of MLPT children.

We further investigated functional coherence by calculating enrichment in curated pathways from Gene Ontology (GO) and Kyoto Encyclopedia of Genes and Genomes (KEGG). As for GO categories, although the number and types of up regulated genes varied widely between the all, male, and female groups, they were all enriched for biological pathways related to immune response and antigen binding. Among them, the placenta of male MLPT infants has the largest number of upregulated genes and the highest enrichment in biological pathways such as leukocyte-mediated immunity and lymphocyte-mediated immunity, while the upregulated genes in the placenta of female MLPT infants were also enriched in pathways related to B cell-mediated immunity and immunoglobulin-mediated immune response (Fig. 3e). For all MLPT infants, the upregulated genes were enriched in biological pathways related to humoral immune response. The downregulated genes in the placenta of male MLPT infants were associated with pathways about multicellular organismal process and secondary metabolic process. In addition, the downregulated genes in the placenta of female MLPT infants were also enriched in pathways related to transmembrane signaling receptor activity, immune response, and cytokine production (Fig. 3f).

As for KEGG pathways, the upregulated genes in the placenta of male MLPT infants were enriched for NF-kappa B signaling pathway, NOD-like receptor signaling pathway, and B cell receptor signaling pathway. In the placenta of female MLPT infants, the downregulated genes were associated with pathways of neutrophil extracellular trap formation, chemokine signaling pathway, etc. (Fig. 3g, h).

### Dysregulated PBA-related genes were identified as potential biomarkers

**Fig. 4:**
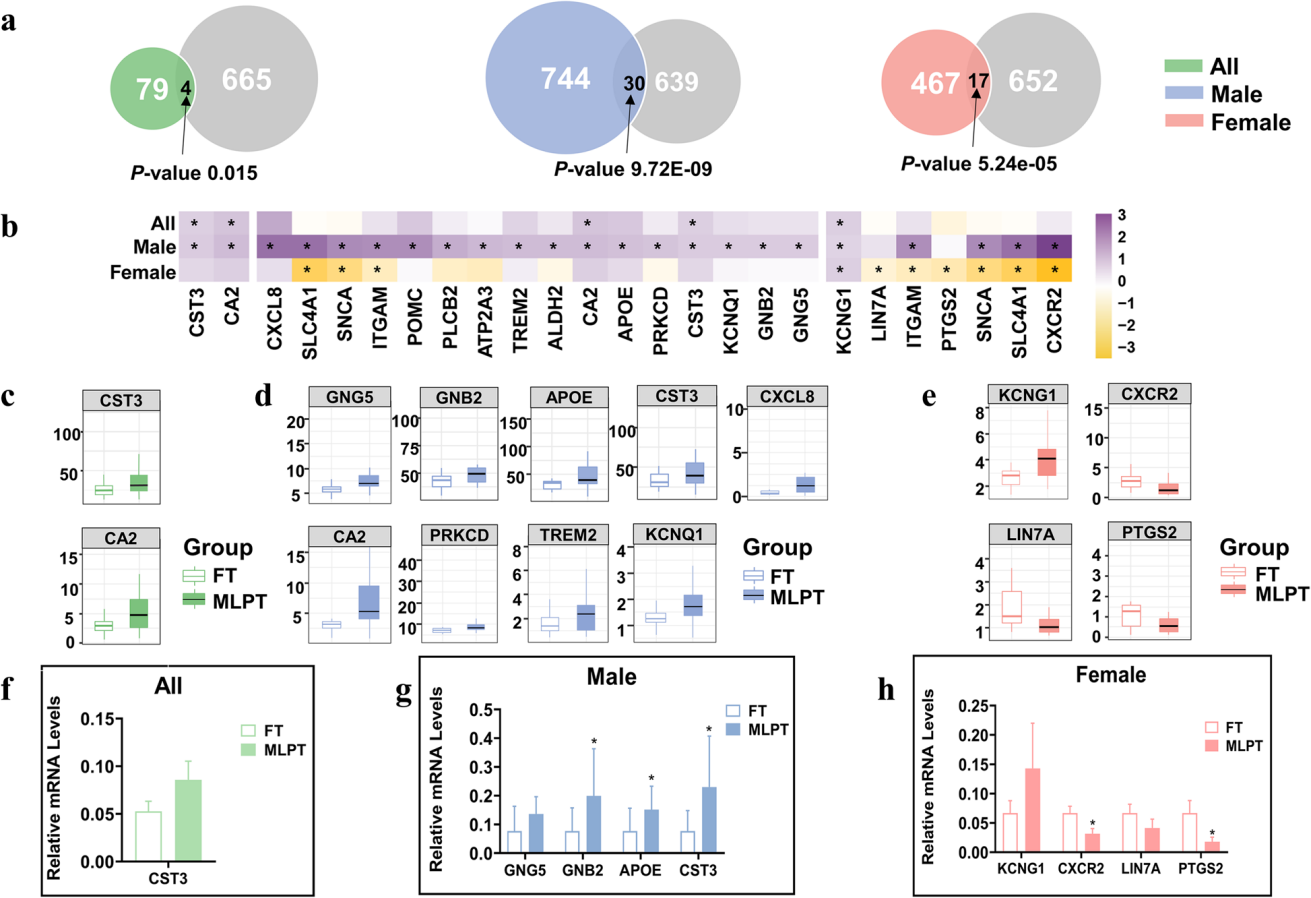
Association between DEGs and PBA-related genes from manual collection and validation of the PBA-related genes in placenta samples from CNBC study. **a** Venn plot of DEGs in MABC study with the PBA-related gene set. The significance of overlap was calculated by hypergeometric test. **b** Heatmap of log2FC for selected PBA-related genes. **c**–**e** Boxplot of FPKM for 14 PBA-related genes. **c** All; **d** male; **e** female. **f** In all placenta samples, *CST3* mRNA expression are not significantly associated with MLPT. **g** In male placenta samples, *GNB2*, *APOE*, and *CST3* mRNA expression are significantly increased in MLPTs. **h** In female placenta samples, *CXCR2* and *PTGS2* mRNA expression are significantly increased in MLPTs. *p < 0.05, **p < 0.01, Mann–Whitney-Wilcoxon test. Error bars indicate SEM

To investigate whether DEGs in MLPT placenta are associated with the increased risk of neurodevelopmental delay in MLPT fetuses, we collected PBA-related gene sets from the literature and related pathways (Additional file 1: Table S6-S7). These PBA-related genes are involved in neurodevelopment pathways, such as synaptic signaling and neuron projection (Additional file 2: Figure S1). Then, we firstly overlapped DEGs with PBA-related genes, and there were 4, 30, and 17 differentially expressed PBA-related genes in the all, male, and female groups, respectively (Fig. 4a). Secondly, to eliminate noise from genes with very low expression levels, we filtered out genes with median FPKM < 1 in both the MLPT and FT groups, leaving 2, 16, and 7 PBA-related genes in the all, male, and female groups, respectively (Fig. 4b). Furthermore, for PBA pathway genes, we retained the genes whose coefficient of variation (CV) were less than 1 in both MLPT and FT group. Finally, there were 14 PBA-related genes (including 12 PBA pathway genes and 2 PBA genes) for subsequent experimental verification (Additional file 1: Table S8), among which 2, 9, and 4 were in the all, male, and female groups, respectively (Fig. 4c–e). Regardless of sex specificity, *CST3* and *CA2* were upregulated in the all group (Fig. 4c). Considering sex specificity, *CXCL8*, *CA2*, *PRKCD*, *TREM2*, *KCNQ1*, *GNG5*, *GNB2*, *CST3*, and *APOE* were upregulated in the male group (Fig. 4d); *KCNG1* was upregulated, and *PTGS2*, *CXCR2*, and *LIN7A* were downregulated in the female group (Fig. 4e). In short, we selected 14 placenta-brain axis-related genes which were also DEGs for further experimental validation considering fold change, FDR and CV (Additional file 1: Table S9).

### Independent validation of the selected biomarkers

We further validated the selected 14 PBA-related genes using RT-qPCR in an independent cohort (CNBC, see “ Methods” section). Regardless of sex specificity, *CST3* was upregulated in the MLPT placentas, which was consistent with the trend in RNA-seq (Fig. 4f). Considering sex specificity, the expression pattern of four RNAs in male placentas and four RNAs in female placentas validated in CNBC from RT-qPCR were all consistent with those in MABC from RNA-seq. Additionally, three (*GNB2*, *APOE*, *CST3*) RNAs in male and two (*CXCR2*, *PTGS2*) RNAs in female and showed statistical significance (Fig. 4g–h). Other PBA-related RNAs’ expression patterns for experimental validation in CNBC cohort were contrary to the expression trend in RNA-seq (Additional file 2: Figure S2).

### Prediction models for neurodevelopmental delay in MLPT children

**Fig. 5:**
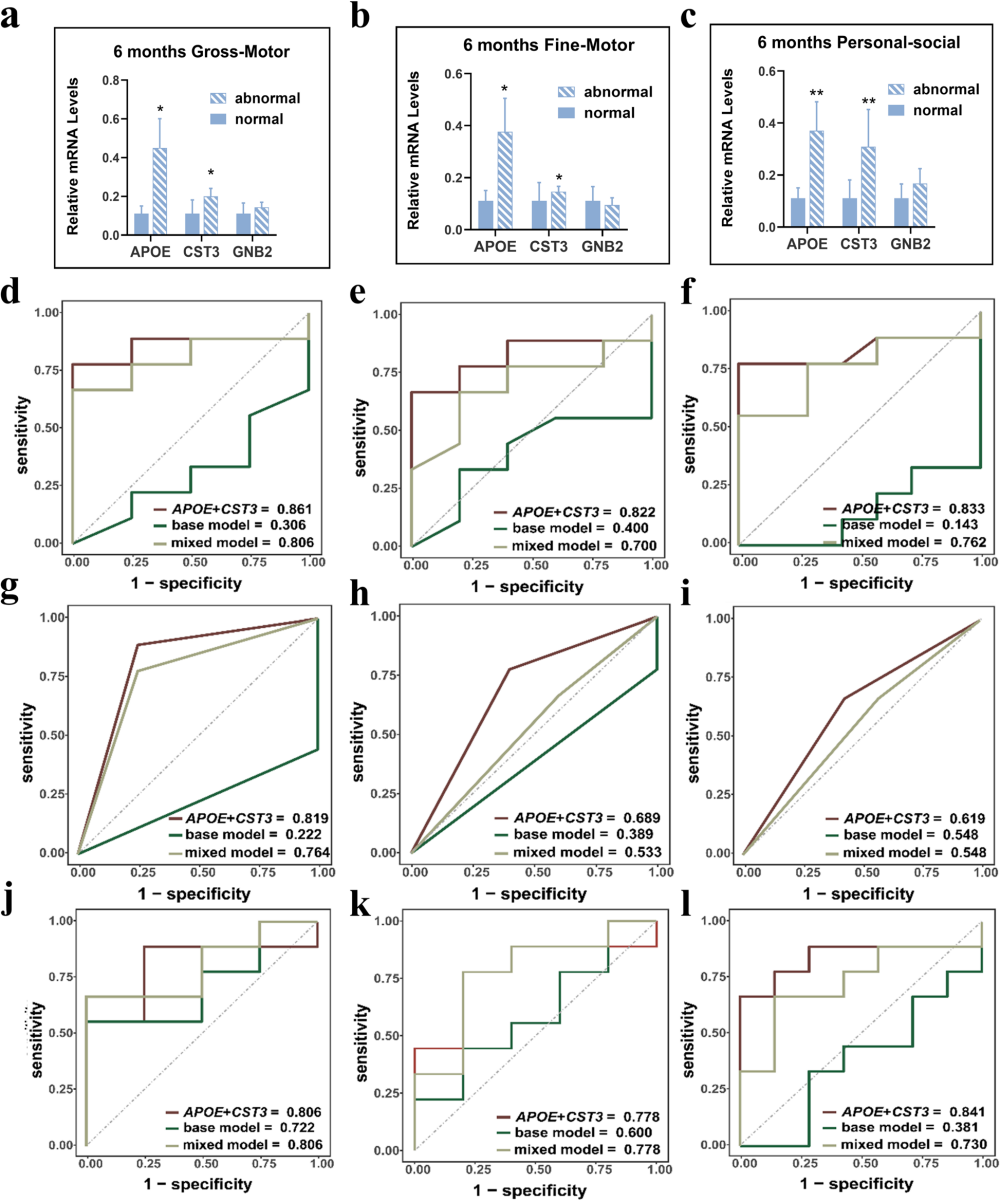
Validation of the three main PBA-related genes in male placenta samples from MABC study and ROC curves of three prediction models for neurodevelopment in MLPT children from MABC study. **a**–**c** For male neurodevelopment delay at 6 months, *APOE* and *CST3* mRNA expression are significantly increased in MLPTs. **a** Gross-motor domain; **b** fine-motor domain; **c** personal-social domain. **d**–**f** ROC curves of prediction model using random forest. **d** Gross-motor domain; **e** fine-motor domain; **f** personal-social domain. Base model: birth weight + gestational age at delivery + maternal age + monthly income family capita + pre-pregnancy BMI, mixed model: *APOE* + *CST3* + base model. **g**–**i** ROC curves of prediction model using logistic regression. **g** Gross-motor domain; **h** fine-motor domain; **i** personal-social domain. Base model: birth weight + gestational age at delivery + maternal age + monthly income family capita + pre-pregnancy BMI, mixed model: *APOE* + *CST3* + base model. **j**–**l** ROC curves of prediction model using decision tree. **j** Gross-motor domain; **k** fine-motor domain; **l** personal-social domain. Base model: birth weight + gestational age at delivery + maternal age + monthly income family capita + pre-pregnancy BMI, mixed model: *APOE* + *CST3* + base model

Next, we performed RT-qPCR to evaluate the performance of the identified signatures for predicting neurodevelopmental delay of male (three RNAs) and female (two RNAs) in MLPT cohort. We found that *APOE* and *CST3* were significantly upregulated in the placentas of MLPT children with developmental delay in gross motor skills domain, fine motor skills domain, and personal-social skills domain at 6 months, which suggested that *APOE* and *CST3* are reliable biomarkers for neurodevelopment prediction of MLPT children (Fig. 5a–c and Additional file 2: Figure S3).

Next, we used three classifiers to access the predictive capacity of *APOE* and *CST3* expression level determined by RT-qPCR. We found that the random forest (RF) model that take both *APOE* and *CST3* as input achieved a higher AUC in predicting the gross motor skills domain (AUC = 0.861), fine motor skills domain (AUC = 0.822), and personal-social skills domain (AUC = 0.833) than model take *APOE* or *CST3* alone, which also outperformed the logistic regression (LR) and decision tree (DT) models based on the same data as a whole (Fig. 5d–l). Adding potentially relevant clinical factors (birth weight, gestational age at delivery, maternal age, monthly income of family per capita and pre-pregnancy BMI) could not further boost model performance. It proved that *APOE* and *CST3* expression levels alone are good predictors (Additional file 1: Table S9).

## Discussion

The intensity and duration of the effect of MLPT on children’s neurobehavioral development remain to be determined. A longitudinal cohort study in Australia found that MLPT children had significantly higher risks of cognitive delay, language delay, motor delay, and social-emotional ability problems at the correction age of 2 years compared with children born at term [31]. A study from China revealed that MLPT children had a high overall developmental delay rate, especially in the fine motor domain (*OR*: 2.43, 95% *CI*: 1.04–5.56) [32]. However, an Italian study using the Bayley-II Scale to evaluate the neural development of infants and young children found that the strength of association between MLPT and neural development did not reach a statistically significant level at the corrected age of 12 and 18 months [21]. Overall, our prospective cohort study indicated that there existed a strong statistically significant association between MLPT and neurodevelopmental delays from 6 months old to 48 months old, which provided obvious evidence for an increased risk of neurodevelopmental delay in children with MLPT.

Furthermore, our data informed that PBA-related genes might be the potential gene targets of MLPT’s impact on fetal neurodevelopment. The placenta acts as an endocrine organ and secretes neuroactive signaling molecules that regulate the function of other endocrine glands to effect infants’ neurodevelopment [33–36]. In our study, we systematically collected the PBA-related gene sets from published articles and related databases, which provided a useful resource for subsequent studies.

Our results suggested that male MLPT infants were associated with more neurodevelopmental delays than females. Similarly, recent studies indicated that there were sex-specific in neurodevelopmental outcomes in preterm infants [37–39]. Male neurodevelopment appeared to be much more sensitive than female to poor postnatal growth, but the specific mechanism still needed to be explored [40, 41]. The above results indicated that both neurodevelopmental outcomes and PBA-related genes are sex-specific in MLPT infants.

It is well known that *APOE* is an important gene in the occurrence and progression of neurodegenerative diseases [42, 43]. In recent years, the role of *APOE* in children’s neurodevelopment has also attracted much attention [44–46]. *APOE* was an important molecule due to not only its lipid transport and cholesterol metabolic functions but also involved in several biological processes, such as response to oxidative stress, cGMP biosynthetic processes, and glutamate receptor clustering [47], which are associated with controlling brain microenvironment homeostasis and partially modulating synaptic plasticity. What is more, lipopolysaccharides (LPS) induced inflammation and decreased the mRNA expression level of *APOE* in both rat and human placentas [48]. In addition to this anti-inflammatory function, placental *APOE* also promotes the transfer of cholesterol from maternal to fetal circulation. *APOE* has been shown to be anti-inflammatory, neuroprotective, and essential for neurodevelopment [49, 50]. *APOE* might work as an important factor for neurodevelopmental disabilities [51]. *CST3* gene encodes an inhibitor of cysteine proteinases, which is widely found in biological fluids and especially in the cerebrospinal fluid that surrounds and protects the brain and spinal cord [52]. The gene’s function is related to the formation of amyloid plaques; thus, it has already been shown to be involved in some brain disorders [53, 54]. Previous studies are showing that *CST3* is strongly expressed in the brain and decidual tissue of the placenta [55–57]. It had been demonstrated that an overexpression of the *CST3* gene in the placenta and higher levels of cystatin C existed in the dopamine-depleted rat striatum [58]. There was a link between these early insults and neurodevelopmental disorders in which there is dopamine dysregulation [27]. Thus, the placental abnormal expression of *CST3* might cause abnormal neurodevelopment in children by dysregulation affecting dopamine. Recently, based on the result of a whole genome methylation analyze of schizophrenia patients [59], *CST3* was considered to be important for neurodevelopment [60]. Besides, some studies suggest that injection of *CST3* into the hippocampus and dentate gyrus or in vitro application of *CST3* played a role in the mediation of neuronal death, and increased expression of *CST3* in glial cells was accompanied by neuronal death [61, 62]. These studies also provide support for our finding that *CST3* can be used as a biomarker for neurodevelopment. However, *APOE* and *CST3* are PBA pathway genes that we screened based on the PBA pathway. These existing studies are not enough to prove that the abnormal regulation of *APOE*, and *CST3* in the placenta will affect the neural development of the fetus. Our study provides clues for this, but further experiments are still needed to reveal the potential mechanism.

Our study has several advantages. First, we elucidate the strength and duration of the effect of MLPT on children’s neurobehavioral development based on a large birth cohort, with prospective cohort studies to reduce recall bias and repeated measures at different months of age to increase confidence. Poor ASQ performance in children at early age has been shown to be sensitive to the diagnosis of ASD, a more severe neurodevelopmental disorder [63]. Therefore, our conclusion has certain guiding significance for the early detection of ASD in children with MLPT. Secondly, we systematically sorted out the PBA-related genes and provided resources for related research. For the first time, we focused on MLPT, rather than the placental transcriptome of all preterm or very preterm children, and revealed that placental gene regulation in MLPT children is aberrant and sex-specific. In the end, the results of this study will serve as evidence for the increased risk of abnormal neurobehavioral development in MLPT children, provide targets for molecular mechanism research, offer biomarkers and intelligent models for risk prediction, and shed light on early management and intervention of MLPT children.

However, our study also has some limitations. First, the sex-specific association between high placental *APOE* and *CST3* expression and increased risk of neurobehavioral developmental delays in MLPT children requires further in vivo and in vitro experiments to reveal the mechanism. In addition, although the PBA-related genes were collected from the literature after rigorous screening, they were not come from the meta-analysis which may weaken the reliability of evidence. Further understanding of PBA-related genes would require a formal systematic review which contains a more global analysis. In the differential expression analysis, we used a significance threshold of FDR < 0.2 for this analysis based on some published literature to avoid missing the potential differences. However, the DEGs identified need further verification to avoid false positives. Due to technical limitations, it is difficult to accurately distinguish maternal and fetal surfaces in our placenta samples. Moreover, because placental tissue is composed of multiple cell types, including stromal cells, mesenchymal cells, and trophoblast cells, biomarker detection may be somewhat heterogeneous [64–66]. What is more, although it is reasonable to assume that the gene expression of *APOE* and *CST3* in placenta will influence the neurodevelopment of infants, the expression trends of *APOE* and *CST3* in the fetal brains may be different from the trends in the placentas, which still needs more studies to explore the regulations of *APOE* and *CST3* by using fetal brain samples. Therefore, the prediction model composed of *APOE* and *CST3* needs to be validated in larger independent cohort.

## Conclusions

This study supports a sex-specific association between MLPT and neurodevelopmental delay in children and demonstrated that the placental-brain axis genes could be used as placental biomarkers to predict the risk of neurodevelopmental delay in boys with MLPT.

### Supplementary Information

**Additional file 1:** **Table S1.** Primers for the RT-qPCR. **Table S2.** Results of generalized estimating equations models between MLPT and ASQ. **Table S3.** Results of multiple testing correction of logistic regression between MLPT and ASQ. **Table S4.** RNA-seq data statistical summary. **Table S5.** List of differential expression genes. **Table S6.** PBA genes. **Table S7.** PBA pathway genes. **Table S8.** PBA related genes for experimental validation in CNBC cohort. **Table S9.** Neurodevelopmental delays prediction and model development.

**Additional file 2:** **Figure S1.** GO functional annotation clustering analysis of PBA related genes from manual collection. **Figure S2.** Validation of the main PBA related genes in placenta samples from CNBC study. **Figure S3.** Validation of the main PBA related genes in MLPT placenta samples from MABC study.

## Data Availability

The RNA sequencing datasets generated and analyzed in the current study have been submitted to the NCBI Sequencing Read Archive (SRA) with accession number SRP410951.

## Abbreviations

ASQ-C: Ages and Stages Questionnaire of China
AUC: Area under curve
BMI: Body mass index
BP: Biological process
CC: Cellular component
CNBC: China National Birth Cohort
DEGs: Differentially expressed genes
DT: Decision tree
GEE: Generalized estimating equations
GO: Gene ontology
LR: Logistic regression
MABC: Ma’anshan Birth Cohort
MF: Molecular function
MLPT: Moderate and late preterm
OR: Odds ratio
PBA: Placenta-brain axis
RF: Random forest
ROC: Receiver operator characteristic
RT-qPCR: Real-time quantitative PCR
