# Supplementary material for: Identification and prediction model of placenta-brain axis genes associated with neurodevelopmental delay in moderate and late preterm children
Source: BMC Med. 2023 Aug 26;21:326. doi: 10.1186/s12916-023-03023-1 (PMC10464496; doi:10.1186/s12916-023-03023-1)
Supplement: Supplementary file 2 — Additional file 2: Figure S1. GO functional annotation clustering analysis of PBA related genes from manual collection. Figure S2. Validation of the main PBA related genes in placenta samples from CNBC study. Figure S3. Validation of the main PBA related genes in MLPT placenta samples from MABC study. [file 12916_2023_3023_MOESM2_ESM.docx]

**Additional file 2**

**Additional figures**

**Figure S1. Gene ontology (GO) functional annotation clustering analysis of PBA related genes from manual collection.**

**
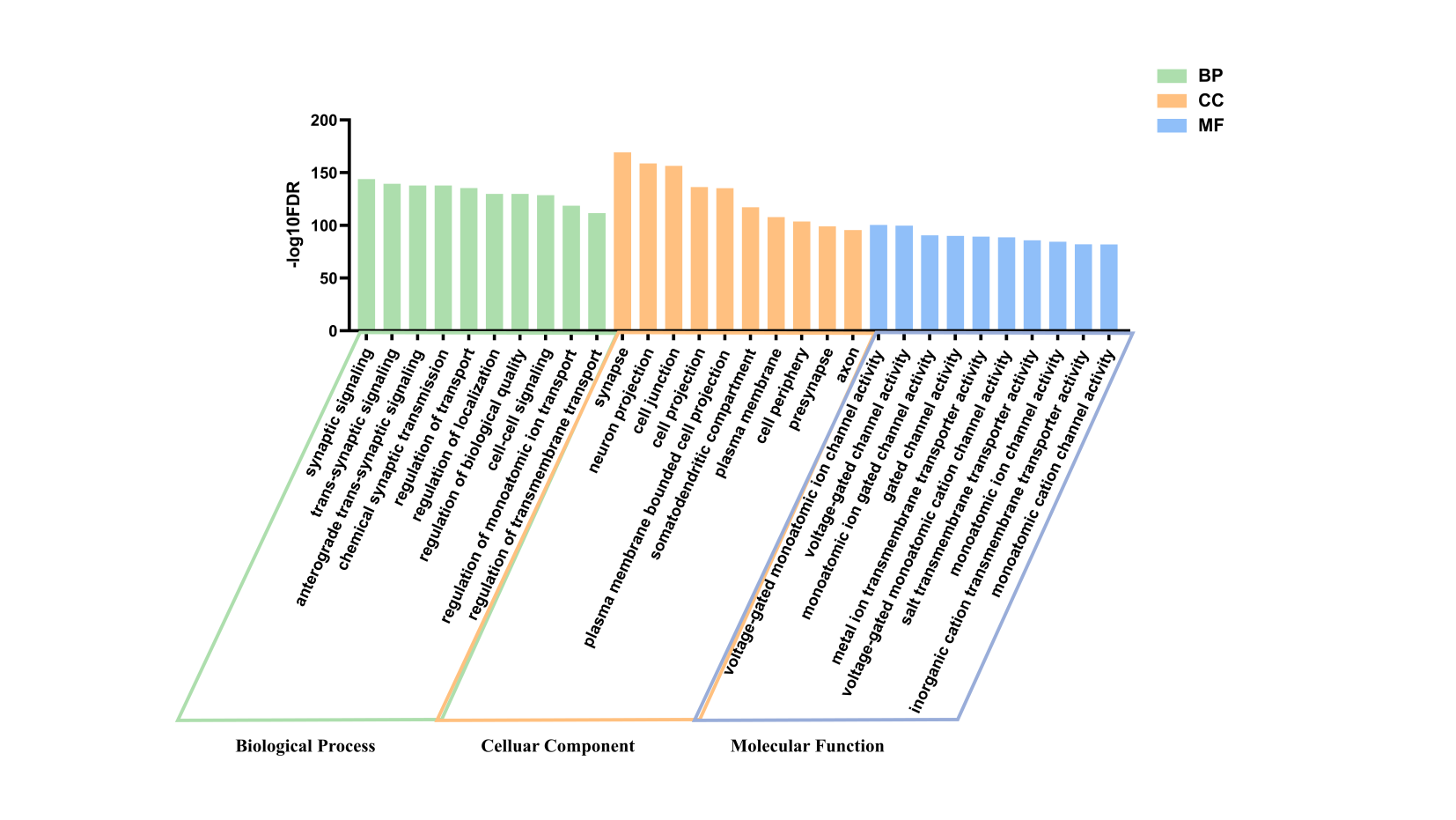
**

**Figure S2. Validation of the main PBA related genes in placenta samples from CNBC study.**


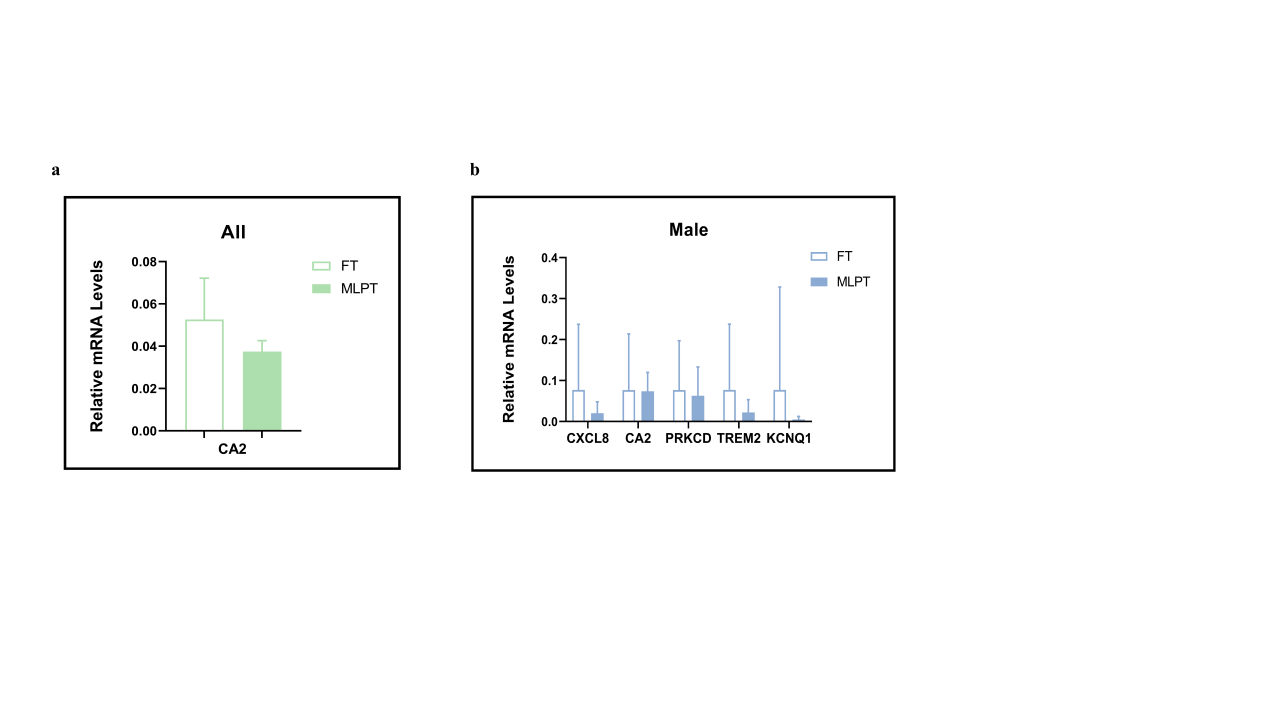


(a) In all placenta samples, *CA2* mRNA expression are not significantly associated with MLPT.

(b) In male placenta samples, *CXCL8*, *CA2*, *PRKCD*, *TREM2* and *KCNQ1* mRNA expression are not significantly associated with MLPT.

**Figure S3. Validation of the main PBA related genes in MLPT placenta samples from MABC study.**

**
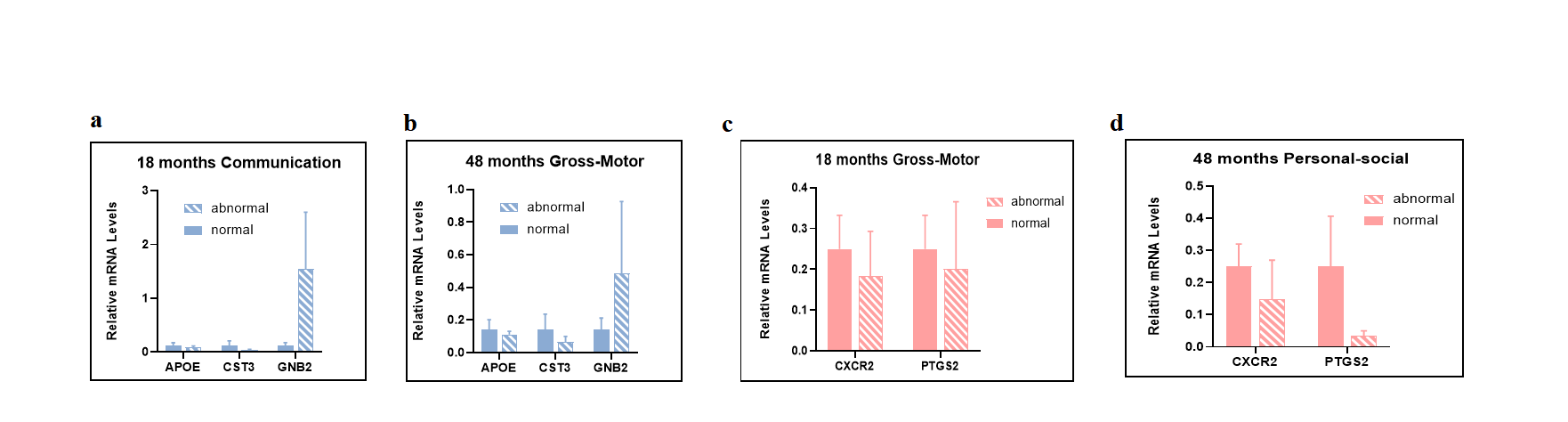
**

(a) For male at 18 months, *APOE*, *CST3* and *GNB2* mRNA expression are not significantly associated with communication domain delay in MLPTs.

(b) For male at 48 months, *APOE*, *CST3* and *GNB2* mRNA expression are not significantly associated with personal-social domain delay in MLPTs.

(c) For female at 18 months, *CXCR2* and *PTGS2* mRNA expression are not significantly associated with gross-motor domain delay in MLPTs.

(d) For female at 48 months, *CXCR2* and *PTGS2* mRNA expression are not significantly associated with personal-social domain delay in MLPTs.
